# Glioma imaging in Europe: A survey of 220 centres and recommendations for best clinical practice

**DOI:** 10.1007/s00330-018-5314-5

**Published:** 2018-03-13

**Authors:** S. C. Thust, S. Heiland, A. Falini, H. R. Jäger, A. D. Waldman, P. C. Sundgren, C. Godi, V. K. Katsaros, A. Ramos, N. Bargallo, M. W. Vernooij, T. Yousry, M. Bendszus, M. Smits

**Affiliations:** 1 Lysholm Neuroradiology Department, National Hospital for Neurology and Neurosurgery, London, UK; 2 Department of Brain Rehabilitation and Repair, UCL Institute of Neurology, London, UK; 3 Imaging Department, University College London Hospital, London, UK; 4 Department of Neuroradiology, University Hospital Heidelberg, Heidelberg, Germany; 5 Department of Neuroradiology, San Raffaele Scientific Institute, Milan, Italy; 6 Neuroimaging Sciences, University of Edinburgh, Edinburgh, UK; 7 Institution for Clinical Sciences/Radiology, Lund University, Lund, Sweden; 8 Centre for Imaging and Physiology, Skåne University hospital, Lund, Sweden; 9 General Anti-Cancer and Oncological Hospital “Agios Savvas”, Athens, Greece; 10 Central Clinic of Athens, Athens, Greece; 11 University of Athens, Athens, Greece; 12 Hospital 12 de Octubre, Madrid, Spain; 13 Image Diagnostic Centre, Hospital Clinic de Barcelona, Barcelona, Spain; 14 Magnetic Resonance Core Facility, Institut per la Recerca Biomedica August Pi i Sunyer (IDIBAPS), Barcelona, Spain; 15 Department of Radiology and Nuclear Medicine, Erasmus MC, Rotterdam, The Netherlands; 16 Department of Epidemiology, Erasmus MC, Rotterdam, The Netherlands

**Keywords:** Brain neoplasms, Magnetic resonance imaging, Surveys and questionnaires, Guideline, Glioma

## Abstract

**Objectives:**

At a European Society of Neuroradiology (ESNR) Annual Meeting 2015 workshop, commonalities in practice, current controversies and technical hurdles in glioma MRI were discussed. We aimed to formulate guidance on MRI of glioma and determine its feasibility, by seeking information on glioma imaging practices from the European Neuroradiology community.

**Methods:**

Invitations to a structured survey were emailed to ESNR members (n=1,662) and associates (n=6,400), European national radiologists’ societies and distributed via social media.

**Results:**

Responses were received from 220 institutions (59% academic). Conventional imaging protocols generally include T2w, T2-FLAIR, DWI, and pre- and post-contrast T1w. Perfusion MRI is used widely (85.5%), while spectroscopy seems reserved for specific indications. Reasons for omitting advanced imaging modalities include lack of facility/software, time constraints and no requests. Early postoperative MRI is routinely carried out by 74% within 24–72 h, but only 17% report a percent measure of resection. For follow-up, most sites (60%) issue qualitative reports, while 27% report an assessment according to the RANO criteria. A minority of sites use a reporting template (23%).

**Conclusion:**

Clinical best practice recommendations for glioma imaging assessment are proposed and the current role of advanced MRI modalities in routine use is addressed.

**Key Points:**

• *We recommend the EORTC-NBTS protocol as the clinical standard glioma protocol.*

• *Perfusion MRI is recommended for diagnosis and follow-up of glioma.*

• *Use of advanced imaging could be promoted with increased education activities.*

• *Most response assessment is currently performed qualitatively.*

• *Reporting templates are not widely used, and could facilitate standardisation.*

**Electronic supplementary material:**

The online version of this article (10.1007/s00330-018-5314-5) contains supplementary material, which is available to authorized users.

## Introduction

Gliomas are a diverse group of neoplasms, the principal treatment for which is surgical resection followed by radiation and/or chemotherapy. Despite ongoing efforts to advance treatments, practically all adult gliomas eventually progress and have an overall poor prognosis [1]. Magnetic resonance imaging (MRI) is fundamental to the characterisation of brain tumours, guides the surgical strategy and is required to monitor treatment response. There is a current lack of MRI protocol standardisation [2], which can be problematic for patient management. Differences in scanning protocols (spatial and contrast resolution, image planes, sequences, etc.), whether within the same institution or between institutions, may affect image interpretation, assessment of contrast enhancement and (volume) changes in follow-up examinations [3]. For advanced imaging modalities, the absence of uniform protocols may delay their implementation, hamper the establishment of threshold values, and in the worst case render the technique non-diagnostic.

In 2015, the Diagnostic Committee of the European Society of Neuroradiology (ESNR) held a workshop on glioma imaging practices at its 38th Annual Meeting in Naples, Italy. Among the audience present, the lack of recommendations for MRI in clinical practice was found to be a universal deficit, whilst variations in protocols seemed to exist.

Best practice is defined as the conscientious and judicious use of current best evidence in making decisions about the care of individual patients [4]. The published evidence around brain tumour MRI protocols constitutes a complex and dynamic entity, particularly where advanced techniques are concerned. Key changes have occurred in the understanding of glioma, which are reflected in the recent World Health Organisation (WHO) classification [5]. It is now clear that the biological aggressiveness of glioma subtypes is primarily influenced by their molecular genetic composition, in some cases discrepant from histological results and conventional imaging features [6–8]. MRI protocols must account for the new integrated approach to glioma classification, and should aim to complement and add value in the diagnostic workup. The goal is to develop imaging protocols, which reflect best practice, but also to consider differences between institutions in equipment, levels of expertise, and financial factors in resource-limited healthcare systems. Furthermore, protocol harmonisation could serve as a means of quality assurance and support multicentre research into new treatments.

Consensus recommendations have recently been developed for glioma imaging in clinical trials. The United States National Brain Tumor Society (NBTS), Society for Neuro-oncology (SNO) and the European Organisation for Research and Treatment of Cancer (EORTC) jointly published the EORTC-NBTS protocol [9]. The main aim of this protocol is to enable in a defined group of patients a reproducible assessment of tumour volume change according to the response assessment in neuro-oncology (RANO) criteria [10]. The focus of this protocol is therefore on anatomical T1-weighted (T1w), T2-weighted (T2w) and T2w fluid-attenuated inversion recovery (T2-FLAIR) sequences, and also includes recommendations for diffusion-weighted imaging (DWI).

The question has been raised whether the EORTC-NBTS protocol would be suitable for implementation in a clinical setting. A simple adoption of a trial protocol into the challenging clinical service may, however, be problematic. In the clinical context, a variable number of questions need to be addressed such as diagnosis, differential diagnosis as well as treatment planning, outcome and monitoring. Furthermore, a clinical protocol must be time efficient and applicable in a wide range of medical institutions, and must affect the management of the individual patient. Advanced techniques, which are not relevant in current clinical trials and therefore not included in the EORTC-NBTS protocol, can be important for patient management.

This paper aims to provide best clinical practice recommendations on conventional and advanced MRI of glioma patients and assesses whether the EORTC-NBTS protocol would be suitable for routine clinical practice. To inform the recommendations and to assess their feasibility, information was sought from European institutions about MRI practices, technical parameters and common diagnostic challenges. To this end, a structured survey was carried out to ensure the involvement and representation of the European neuroradiology community in the guidance.

## European survey on glioma MRI practices

### Method

An online questionnaire was designed using a Google forms open access toolbox (Google.com, Mountainview, CA, USA). The questionnaire featured 87 items, divided into multiple choice, single best choice and free text questions on personal practice, preferred MRI techniques and clinical scenarios (see online Supplement 1). The questionnaire was optimised and tested by peers such that it would take a maximum of 10 min to fill out. This information was given at the start of the questionnaire.

Questions were derived from issues raised at the 38th ESNR annual meeting workshop on brain tumour imaging (attendance ± 150 people), as well as those identified during the development of the EORTC-NBTS protocol. Survey invitations were emailed to all ESNR members (n=1,662), non-members who had expressed their interest in ESNR-activities in the past (n=6,400), European national neuroradiological societies (The Netherlands, Belgium and the UK), and distributed via LinkedIn and Twitter. The survey was open for 1 month, from 1 March to 1 April 2016, with one reminder sent. To avoid duplicate bias, participants were instructed to supply institution details or confirm they were the only person answering from their centre.

### Results

#### Demographic and institution data (online Supplement 2, Table 1)

**Fig. 1:**
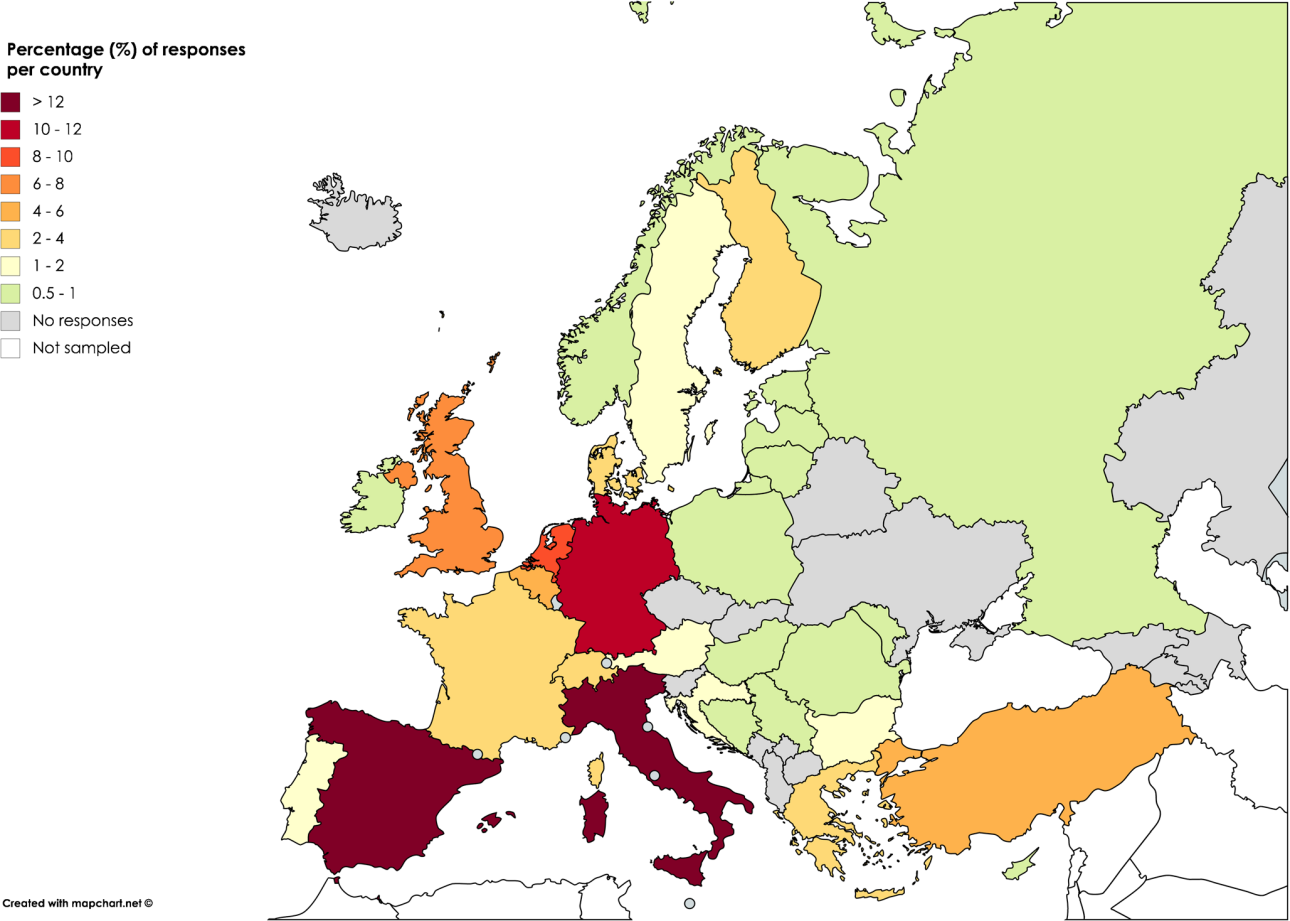
Institutional responses (%) per country. Countries with no responses are shaded grey

Two hundred and twenty-seven professionals working in 31 out of 51 European countries completed the survey; seven were duplicates from the same institution, resulting in responses from 220 institutions. A proportion of questionnaires (8.2 %) included in the analysis were submitted by individuals currently working outside Europe. Figure 1 provides an overview of the responses by country.

A number of questions included the option ‘other’. If this was answered by < 5 % of individuals, percentages are not quoted in the results. A few undecipherable free text answers were excluded from the analysis.

#### Primary diagnosis and follow up (online Supplement 2, Table 2)

**Fig. 2:**
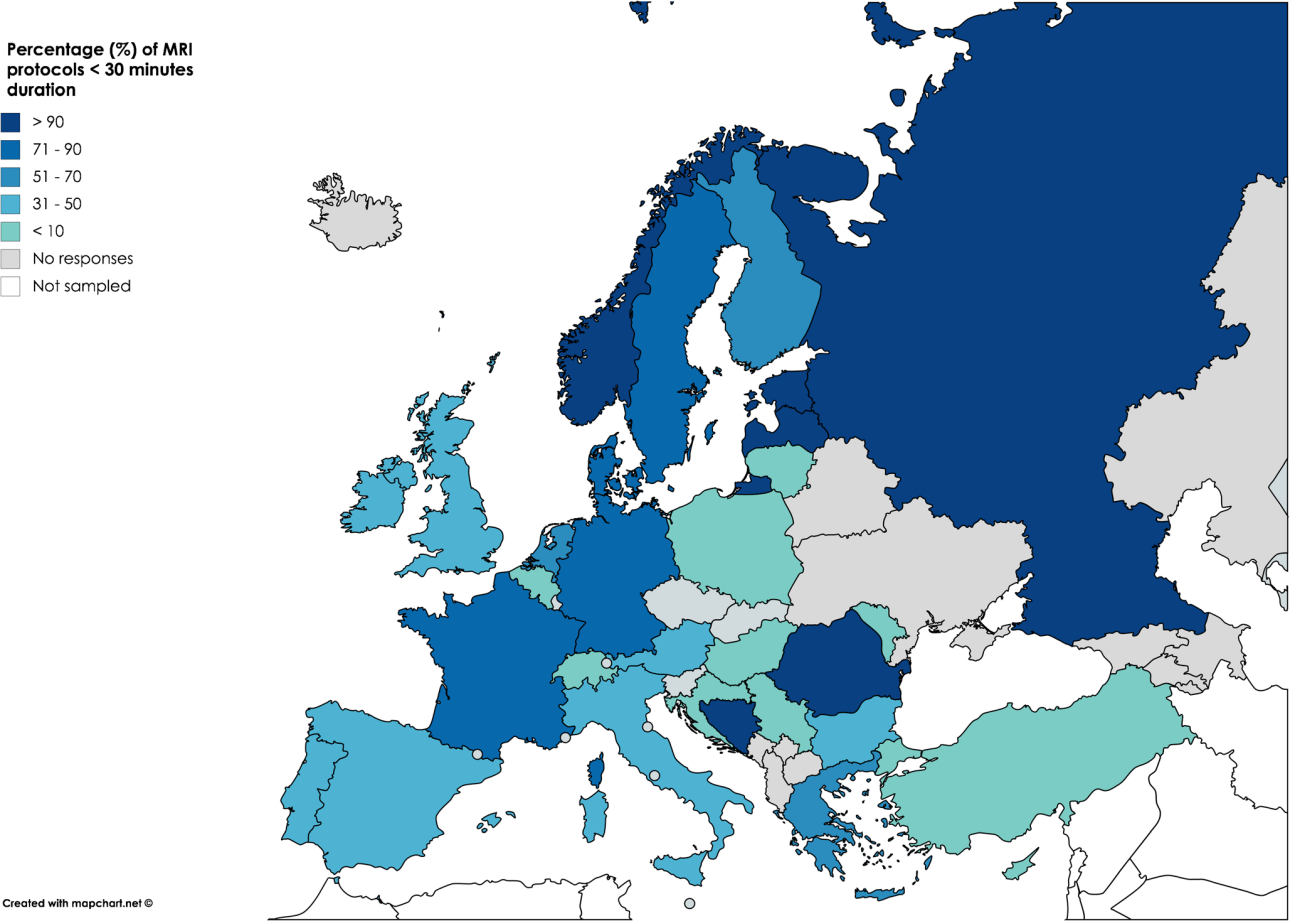
Percentage of MRI protocols of < 30 min duration for each country. The remaining protocols nearly always lasted between 31 and 60 min. Amongst all 220 responses, only 2.7 % of glioma MRI protocols were longer than 60 min

Typical glioma standard MRI protocols lasted between 20–60 min. The proportion of institutions per country that use protocols shorter than 30 min is displayed in Fig. 2.

In more than 95 %, the protocols included T2w, T2-FLAIR, pre- and post-contrast T1w, and DWI. At many institutions (65 %) T2*w or susceptibility weighted imaging (SWI) were part of the MRI protocol. 3D anatomical sequences were used by most (81.8 %) of the institutions, most commonly post-contrast T1w. In free text answers, reasons given for not using 3D imaging included time pressure (n=10), quality concerns, scanner limitations, financial reasons and lack of technical support (n=2 each). Most (77.7 %) institutions used the same protocol for glioma follow-up as for primary diagnosis. Some chose a different protocol for follow up with omission or selective use of sequences, most frequently MR spectroscopy (MRS) or perfusion MRI (pMRI).

#### Contrast-enhanced MRI (online Supplement 2, Table 3)

To depict enhancement, the most commonly (72.3 %) performed sequence was FSPGR/MPRAGE. Not all users felt comfortable using this 3D gradient echo sequence as the sole sequence to assess contrast uptake. In free text answers, the most frequently (n=48) reported concern was absent or suboptimal sensitivity to detect enhancement, followed by artefact, reduced sensitivity, and a risk of missing small lesions.

#### Diffusion-weighted imaging (DWI; online Supplement 2, Table 4)

DWI was almost always (99.1 %) performed in glioma imaging. ADC was much more often (78.2 %) assessed by visual comparison with normal appearing brain than quantitatively. Nearly all users employed b values of 0 and 1,000 s/mm^2^, with some acquiring an additional b-value of 500 s/mm^2^.

#### Perfusion MRI (pMRI; online Supplement 2, Table 5)

Most institutions (85 %) applied pMRI (most frequently dynamic susceptibility contrast [DSC; 81.8 %]) for initial grading and/or glioma follow-up. The use of this modality was homogeneously distributed across Europe. Some institutions (21.4 %) reported use of either DSC plus one other perfusion technique, and rarely all three were acquired. Free text answers highlighted usefulness of pMRI in differentiating chemoradiation effects from tumour progression (n=55) and for grading (n=36).

#### MR Spectroscopy (MRS; online Supplement 2, Table 6)

The majority (80.4 %) of institutions used MRS in clinical brain tumour imaging, but rarely as part of the routine protocol. The largest group (35.2 %) of users acquired MRS occasionally, upon request or for a specific indication. Free text answers regarding MRS indications featured lesion characterisation (n= 56), including distinction of tumour from non-neoplastic conditions, and grading (n=21). Less commonly MRS was employed for brain tumours in general or to differentiate therapy effects from tumour recurrence.

#### Functional MRI (fMRI; online Supplement 2, Table 7)

Approximately half (49.8 %) of participating institutions used fMRI in clinical practice, mostly for surgical planning (95.4 %). Free text answers on the clinical impact reported its value for operative planning, to guide the interventional approach, and to determine tumour resectability. Functions assessed were language lateralisation and localisation, visual cortex localisation and motor cortex localisation (resting state fMRI not assessed). fMRI scan times varied substantially lasting up to 1 h, depending on tasks.

#### Diffusion-tensor imaging (DTI) tractography (online Supplement 2, Table 8)

Nearly two-thirds (63.7 %) of participating institutions carried out DTI tractography in their practice, generally for presurgical evaluation (88.2 %). Numerous free text answers stated that DTI tractography was useful for operative planning, underscoring the potential of DTI results to change the surgical approach. Some users reported limited impact or experience. The number of acquired directions varied significantly, but over half (58.5 %) of the DTI performing institutions acquired at least 20 directions in their clinical practice.

#### Clinical scenarios and issues

##### Early postoperative MRI (online Supplement 2, Table 9)

At the majority (74.3 %) of institutions, early postoperative MRI was routinely performed to assess the extent of glioma resection, but few (17.2 %) radiologists provided a percent measure on completeness of resection in their report, with no uniform method identifiable from the free text answers (n=28).

##### Monitoring of therapy response (online Supplement 2, Table 9)

For glioma follow-up, most respondents (60.6 %) undertook a qualitative assessment, and a smaller group (27.1 %) obtained measurements according to the RANO criteria [10]. Less than a quarter (23.3 %) of institutions incorporated a reporting template in their current practice.

##### Post-processing and non-use of advanced imaging (online Supplement 2, Table 10)

For all advanced modalities, data post-processing was most commonly carried out by a radiologist. Multiple reasons featured amongst non-use of advanced imaging, with lack of MRI equipment (40.5–49.5 %) slightly dominating for all methods.

## Discussion

A conventional MRI protocol consisting of T2w, T2-FLAIR, DWI and pre- and post-contrast T1w appears representative of standard glioma imaging practice in Europe. To the best of our knowledge, level I evidence in the form of randomised controlled trials for the MRI assessment of glioma is currently lacking. Conventional MRI, but also the use of DWI and pMRI for glioma characterisation are supported by some level II evidence, and by numerous level III studies [11]. The current data on the use of MRS, SWI, fMRI and DTI are restricted to level III evidence, mostly in the form of retrospective comparative studies.

### 3D versus 2D imaging

3D (volumetric) imaging has clear advantages over 2D imaging. First, reconstructions can be made in all planes, allowing not only for a better appreciation of anatomical location, but also for more accurate longitudinal assessment [12]. Second, tumour volumes can be more accurately measured, in particular when this is done automatically [13]. In addition, the higher through-plane resolution of 3D imaging reduces the risk of missing small foci of contrast enhancement due to partial volume effects [14]. FSPGR/MPRAGE appears overall diagnostic for glioma imaging [15] and remains the most widely available T1w 3D technique at present [9]. However, some concerns exist regarding its suitability to depict post contrast enhancement, increased susceptibility to movement or pulsation artifacts, and lack of sensitivity for the detection of leptomeningeal disease. The short repetition times used for FSPGR/MPRAGE sequences are known to result in less marked T1-dependent signal enhancement compared with spin-echo using the same Gadolinium-chelate dose [16]. This effect was not found to be detrimental for small brain lesions in vivo [17], but potentially superior 3D spin-echo alternatives such as SPACE and CUBE [14, 18] could well supersede FSPGR over time.

### Diffusion-weighted imaging

DWI with a maximum b value of 1,000 s/mm^2^ matches the EORTC-NBTS protocol and National Cancer Institute (NCI) - International Society for Magnetic Resonance in Medicine (ISMRM) consensus recommendations [19], which specify the preferred use of 3 b-values (0, 500 and 1,000 s/mm^2^), but acknowledge the fact that not all scanners have this capability. DWI can non-invasively contribute to estimating tumour cellularity and grade [11, 20–22] and support the assessment of therapy response, although as a single modality its accuracy appears limited for the distinction of tumour and radiation effects [23, 24]. Advanced diffusion techniques could provide greater information on tissue microstructure for the distinction of glioma molecular subgroups [25, 26, 83] and to support early response assessment, e.g. via parametric mapping [27–29], but such methods are not yet a clinical standard.

### T2*w and SWI

Susceptibility sensitive sequences may identify haemorrhage or calcification in glioma primary diagnosis, and help depict biopsy tracts. For SWI, an association has been observed between intratumoral susceptibility signals (ITSS), histological WHO grade and relative cerebral blood volume (rCBV) [30]. The latter could provide substitute evidence of neovascularity, where pMRI is unavailable. However, current evidence is confined to a limited number of studies [31]. It remains doubtful what information SWI can offer above other MRI sequences in glioma, with a possible exception of tumour margin delineation on contrast-enhanced SWI [32].

### Perfusion MRI (pMRI)

DSC pMRI constitutes the primarily used perfusion method (>80 %) in the European institutions surveyed, which matches published data [33], with nearly half of all users acquiring it for all glioma indications. With DSC, high-grade glioma can be differentiated from low-grade glioma using rCBV values with high (95 %) sensitivity, but specificity is relatively low (70 %) [34, 35]. This finding can be attributed to the misclassification of low-grade gliomas with elevated rCBV, most notably oligodendroglioma [36, 37]. Raised rCBV has recently been highlighted as a characteristic of isocitrate dehydrogenase (IDH) wildtype glioma, even at a histological low grade [38]. Furthermore, rCBV is the most validated perfusion parameter for the distinction of therapy effects from tumour progression [39, 40]. DSC studies consistently demonstrate that rCBV is low in areas of radiation necrosis or pseudoprogression and high in tumour progression, allowing for accurate (generally accuracy >90 %) distinction between these entities [41–44].

Alternative perfusion techniques such as dynamic contrast-enhanced (DCE) perfusion MRI and arterial spin labelling (ASL), though less established, appear beneficial, especially for such gliomas in which susceptibility effects render DSC non-diagnostic. Neither technique has, however, been extensively validated or integrated into clinical glioma imaging practice to date.

### Spectroscopy (MRS)

Whilst a high number of institutions have experience with MRS in glioma, the survey results suggest that this method is clinically used for specific indications only. The relative intensity of metabolite spectra is influenced by echo time (TE), with short or intermediate TE (30–144 ms) considered preferable for glioma imaging. The benefit of MRS in the distinction of glioma from non-neoplastic conditions was highlighted in many free text answers, which is well supported by published data [45, 46]. The evidence for the selective use of MRS in the distinction of glioma from other tumours, such as metastases and brain lymphoma, remains indeterminate [47, 48]. A potential advantage of MRS lies in the characterisation of grade II oligodendroglioma, which commonly show elevated rCBV, and may be misclassified as high-grade tumour [49]. Otherwise, MRS finds a less prominent application in grading, tumour classification, biopsy planning and characterisation of radiation effects, with a moderate performance shown for the latter indications in research [50–52]. For glioma grading, Cho/Cr and Cho/NAA ratios have most frequently been reported to increase diagnostic accuracy, but in isolation MRS remains inferior to rCBV measurements [47, 53]. For various thresholds, quantitative MRS suffers from a mismatch between sensitivity and specificity, therefore a clear diagnostic benefit in grading has only been shown through combination with other techniques [34, 50, 53]. For the differentiation of radiation necrosis and recurrent glioma, a systematic meta-analysis revealed a limited performance for MRS, and strongly recommended its use only in combination with other modalities [51].

### fMRI and DTI tractography

With a principal clinical application of surgical planning, these modalities are used to determine language lateralisation and localisation of the motor and visual cortex as well as various white matter tracts. Even though there is now substantial literature support for the use of task-based fMRI in glioma in the pre-operative context, reported accuracies for this modality are variable and its impact on clinical practice remains to be further established [54, 55]. Whilst the notion that DTI may change the surgical approach is supported by data [56, 57] and by the survey results, there are still important limitations to the standardisation and clinical integration of tractography for neurosurgical decision-making [58]. DTI was the only advanced imaging method for which users specifically mentioned limited experience, which is judged to reflect its partial clinical establishment in Europe. Although it has been highlighted that the reliability of DTI may increase according to the number of diffusion directions acquired [57], no consensus was identifiable from the survey results on an optimal number of directions for clinical glioma imaging.

### Non-use of advanced imaging

The fact that data processing for all advanced techniques was most commonly carried out by a radiologist suggests that these methods can be user-led. Such an arrangement could however impact neuroradiology workflow. Lack of MRI facility/equipment or software appears to be a greater limiting factor than time pressure. In some countries, advanced techniques are not reimbursed, which can be a significant hurdle. Lack of experience with and not knowing how to implement the techniques appear to be important obstacles, especially for fMRI.

### Reporting practices and quantification

With improved outcomes after complete or near-complete glioma removal, postoperative residual measurement can be expected to become a focus of attention [59, 60]. Yet most radiologists do not offer any quantitative information in their report, and the literature provides no established system to assess extent of resection.

In follow up, most respondents relied on a visual estimate of tumour size despite existing RANO guidance. The current RANO criteria incorporate two-dimensional measurements, reflecting evidence indicating that changes in tumour volume correlate with changes in unidimensional or two-dimensional measurements [61–63], especially in high-grade glioma [64, 65]. The debate about whether volumetric glioma measurements would be more accurate than linear measurements and/or would impact clinical management is ongoing, especially for the response assessment of lower grade gliomas, which may be challenged by subtle growth. Conflicting results exist regarding the reliability of low-grade glioma segmentations [66, 67]. An additional hurdle is that currently available semi-automated volumetric segmentation algorithms tend to require manual editing [67–70].

An important issue revealed by the survey is the limited use of quantification methods for physiological parameters such as ADC and rCBV. Lack of available software tools and/or familiarity with how to use them, as well as time pressure may be contributing factors to the limited quantification of findings [22, 71].

### Survey limitations

From all persons contacted, only a small proportion (14 % of ESNR members) responded, meaning the survey results may not represent the entire neuroradiology community, almost certainly introducing a response bias from those with a particular interest or expertise in glioma imaging. Moreover, it is likely that the length of the survey contributed to the low participation rate. Duplicate bias was avoided through only allowing one person answer from each institution. The disadvantage of this approach is that variations in practice within one department may not have been captured. This survey does not cover most of the practices in outpatient general radiology outside neuroradiology. We did not survey imaging practices specific to paediatric glioma. However, a central imaging review has been instituted for more than 20 years for paediatric brain tumour studies and recommendations on imaging and response assessment do exist [72, 73].

### Best clinical practice recommendations

**Fig. 3:**
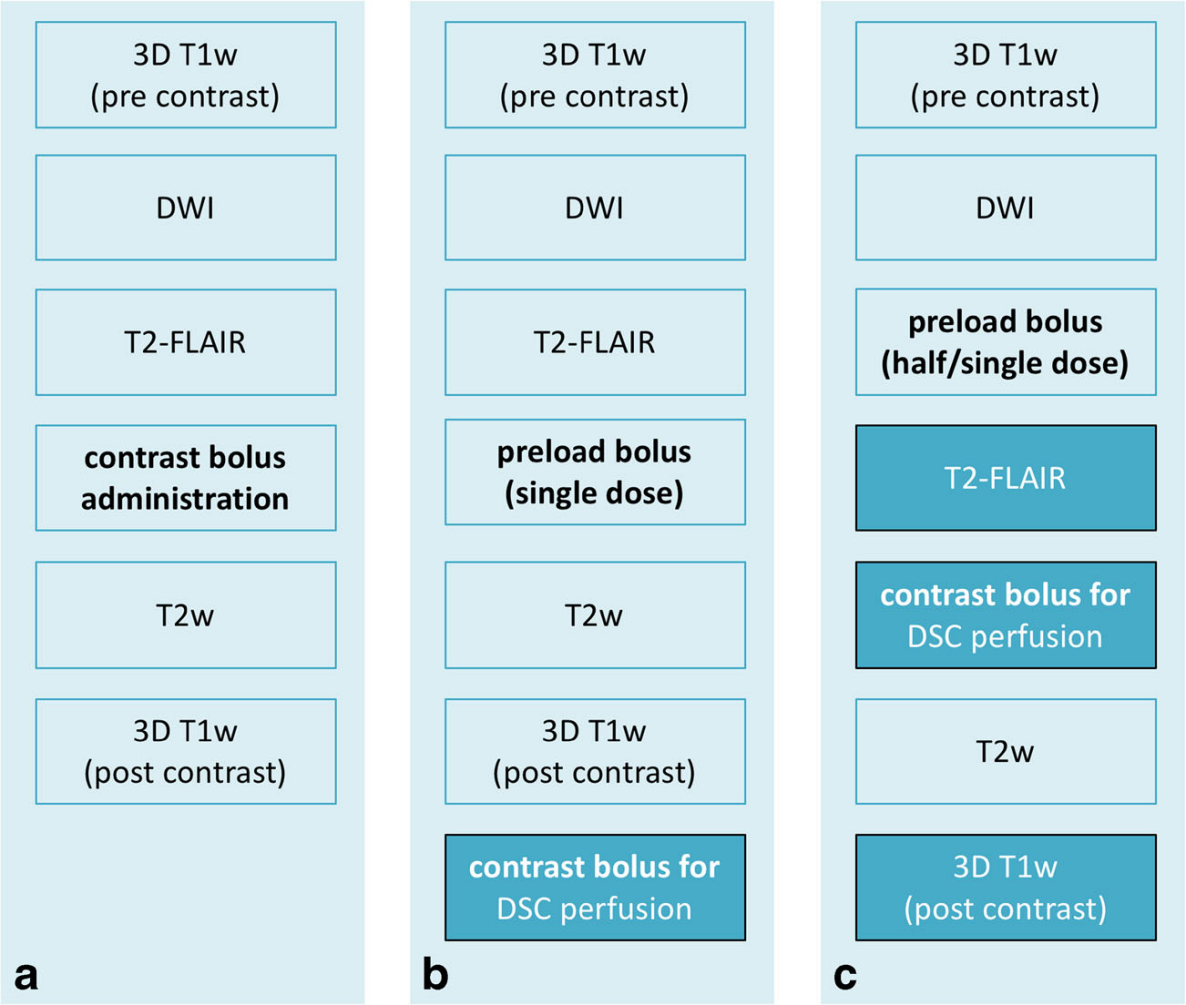
Three possible options for a glioma imaging protocol in clinical practice based on the EORTC-NBTS protocol (**a**), with the addition of DSC perfusion imaging (**b, c**). Option C has the advantage over option B that it has double the contrast dose for post-contrast T1w imaging. Option B may be preferred if non-contrast enhanced T2-FLAIR is desired. Please see Ellingson et al. [9] for further considerations and vendor-specific sequence details on structural and diffusion-weighted imaging. The moment of contrast administration is indicated in bold

The following recommendations for MRI of glioma were formulated taking together the information provided by a peer group (>150 persons) discussion at the 38th ESNR Annual Meeting in 2015, a structured survey of clinical practices at over 200 European hospital institutions from 31 European countries, and the currently available literature on the subject (Fig. 3).

### Conventional MRI protocol recommendations

The MRI sequences prescribed by the EORTC-NBTS protocol are widely used and scan durations generally allow for the implementation of the 25–30 min EORTC-NBTS basic protocol in routine clinical practice. It is therefore recommended that this should be used as a minimum clinical standard. As a base structure this will support glioma imaging standardisation across Europe with a view to establishing databases, which could be shared for radiomics and radiogenomic analyses and upon which advanced techniques can further build in the future.

3D imaging is preferable for the aforementioned reasons and to support the transition into volumetric tumour measurements, but it is recognised that further development is required in this area. Where 3D T1w imaging is adopted, this should be performed as isotropic sequences before and after contrast, taking care to ensure consistent and sufficient post contrast timing [9]. FSPGR/MPRAGE remains the most widely available T1w 3D technique as part of the standard MRI vendor Alzheimer’s Disease Neuroimaging Initiative (ADNI) protocol [74] and is recommended for clinical brain tumour trials [9]. Its use is again endorsed here as an accessible method for serial glioma imaging in clinical practice, but it could be replaced by 3D spin-echo T1w imaging where this is available, or supplemented with 2D spin-echo sequences where optimisation of the 3D technique fails to be maximally sensitive to contrast enhancement. In individual circumstances where only 2D imaging achieves good quality imaging within a clinically justifiable time, this may be retained as a standard.

For diffusion analysis, quantitative ADC comparison to normal brain is recommended, due to the potential pitfall of visual assessment that a tumour surrounded by oedema will appear dark on the ADC map, even in the absence of diffusion restriction. Because of the limited number of studies on T2*w/SWI, these sequences are considered optional. We would suggest using the same anatomical protocol for both primary diagnosis and follow-up to maximise comparability.

### Advanced imaging recommendations

Perfusion MRI should be performed in gliomas of suspected low grade that have not undergone histological evaluation or prior to biopsy [34, 35, 39, 75]. The use of perfusion for serial lesion assessment is recommended to identify malignant transformation and to distinguish therapy effects (pseudoprogression or radiation necrosis) from tumour progression [41–44]. A caveat must be made that threshold values are not simply transferable between institutions, as they very much depend on scan parameters and post-processing methods [76]. Based on currently available data, we recommended DSC as the standard technique. Using pMRI routinely in all glioma patients has several advantages: diagnostic information is available when needed, there is consistency of imaging protocols, and both radiographers and radiologists gain and sustain experience with the technique. The available evidence strongly supports the use of a preload bolus technique, to overcome errors in estimation cerebral blood volume due to contrast leakage effects [40, 77, 78]. Gadolinium contrast dose can – at 3.0T – be kept low by splitting a single dose into preload and bolus injection, as outlined by the American Society of Functional Neuroradiology (ASFNR) in 2015 [40]. The acquisition of an appropriate baseline prior to contrast injection, high temporal resolution (TR<1,500 ms), and fast contrast bolus injection (preferably with a power injector) are important aspects of appropriate DSC acquisition [79]. Consistency of acquisition and post-processing techniques is critical, as differences between software packages, and even algorithm alterations within the same product may produce significantly different quantitative perfusion results [78]. Where sufficient evidence has been gathered within an institution to show the reliability of an alternative technique (DCE, ASL), this could be performed optionally, preferably as an adjunct.

On the basis of the survey results and current data, MRS is recommended in glioma as an optional modality for specific indications as aforementioned. Its clinical indication should be considered on an individual case basis, whereby caution is advised regarding the use of MRS in isolation for some of its less certain indications.

Because of their limited availability and limited use by the survey respondents at the present time, fMRI and DTI will not form part of these recommendations. The authors would like to highlight their potential value, however, and would support their use where adequate facilities, expertise and quality assurance measures exist. Further research into these techniques is desirable and recommended.

### Discussion of recommendations

Relatively easy adaptation towards the standard best clinical practice recommendations can be expected, although some variations throughout Europe are likely to remain, depending on reimbursement strategies, practical and logistical setups and availability of scanning facilities.

For advanced techniques, lack of facility, software and experience is likely to hamper their introduction at some institutions, and it is possible that this could be especially the case for centres from which no survey results were available. The neuroradiological community has an important role here to include such technical aspects in their various training programmes.

For anatomical MRI, the use of measurements, bidirectional as a standard (and optionally volumetric, where segmentation software is available) is strongly recommended, as this has been shown to increase diagnostic accuracy in serial follow up [80]. The use of RANO criteria in clinical practice could be facilitated by the introduction of structured reports, which could also have other advantages both in terms of accuracy and effectiveness [81, 82]. These are preferably developed together with treating physicians, to ensure that all relevant information is consistently reported.

Regarding advanced imaging, we would like to emphasise that, where possible, quantification is a powerful tool in clinical practice, since it allows for the formulation of threshold or reference values and avoids certain pitfalls of subjectivity. Validation is, however, required. Multicentre research will be of key importance to establish transferable quantification methods for advanced imaging, which would be applicable across scanners and vendor platforms.

## Conclusion

The MRI sequences prescribed by the EORTC-NBTS protocol are well established in glioma imaging practice throughout Europe, and we recommend that this protocol is adopted as the clinical standard for anatomical MRI. Advanced imaging methods may offer crucial diagnostic information, and should be utilised where possible, within the constraints of currently available data and local expertise. The results from the literature review and survey highlight the value of pMRI in glioma, and also potentially important roles for other methods. The relative lack of quantitative assessment and reporting templates reflects a further need for standardisation. The harmonisation of glioma imaging protocols across Europe together with ongoing research should aim to support the development of quantitative biomarkers for brain tumour diagnosis and therapy response assessment.

## Electronic supplementary material

ESM 1 (DOCX 81.8 kb)

ESM 2 (PDF 149 kb)

## Abbreviations

ADC: Apparent Diffusion Coefficient
ADNI: Alzheimer’s Disease Neuroimaging Initiative
ASFNR: American Society of Functional Neuroradiology
ASL: Arterial Spin Labelling
Cho: Choline
Cr: Creatine
DCE: Dynamic Contrast-enhanced
DSC: Dynamic Susceptibility Contrast
DTI: Diffusion Tensor Imaging
DWI: Diffusion Weighted Imaging
EORTC: European Organisation for Research and Treatment of Cancer
ESNR: European Society of Neuroradiology
FLAIR: Fluid Attenuated Inversion Recovery
fMRI: Functional MRI
FSPGR: Fast Spoiled Gradient Echo
IDH: Isocitrate Dehydrogenase
ISMRM: International Society for Magnetic Resonance in Medicine
ITSS: Intratumoral Susceptibility Signals
MPRAGE: Magnetization Prepared RApid Gradient Echo
MRI: Magnetic Resonance Imaging
MRS: MR Spectroscopy
NAA: N-acetylaspartate
NBTS: National Brain Tumor Society
NCI: National Cancer Institute
pMRI: Perfusion MRI
rCBV: Relative Cerebral Blood Volume
RANO: Response Assessment in Neuro Oncology
SNO: Society for Neuro-oncology
SPACE: SPAtial and Chemical Shift Encoded Excitation
SWI: Susceptibility Weighted Imaging
WHO: World Health Organisation
