# Supplementary material for: Glioma imaging in Europe: A survey of 220 centres and recommendations for best clinical practice
Source: Eur Radiol. 2018 Mar 13;28(8):3306–17. doi: 10.1007/s00330-018-5314-5 (PMC6028837; doi:10.1007/s00330-018-5314-5)
Supplement: Supplementary file 1 — (DOCX 81.8 kb) [file 330_2018_5314_MOESM1_ESM.docx]

**Supplement 1
ESNR Survey on glioma imaging practices in Europe – list of questions**

**Position**

- Which best describes you?

**Country**

- Which country do you currently practice in (drop down menu Europe)?

**Other country**

- Please specify the country you're currently practicing in:

**Institution**

- Which best describes your current institution?
- Please indicate which specialist services are available at your institution?
- Which of the following describes MRI physics support at your institution?

**Patients**

- For primary brain tumour diagnosis, how many MRI scans do you perform at your institution per week?
- For primary brain tumour follow-up, how many MRI scans do you perform at your institution per week?

**Primary diagnosis of glioma**

- How long does your MRI protocol typically last?
- Which conventional sequences do you routinely perform?
- In addition to the conventional imaging, the protocol routinely includes:
- Do you use 3D acquisition?

**3D acquisition**

- Which sequences do you routinely acquire 3D?

**No 3D acquisition**

- Do you wish to acquire 3D sequences?

**Wishing 3D acquisition**

- Which sequences do you wish to acquire 3D?
- Please describe the obstacle(s) or reason(s) why you're not acquiring 3D sequences at the moment:

**Follow-up imaging**

- Do you use the same imaging sequences for glioma follow-up as for primary diagnosis?

**Follow-up protocol**

- Please specify how your follow-up protocol is different from the protocol you use for primary diagnosis:

**Post-contrast imaging**

- Which T1w post contrast sequence(s) do you perform to assess lesional enhancement?
- Do you feel comfortable with only performing 3D SPGR/MPRAGE (and no spin-echo) imaging to assess post-contrast enhancement?

**3D post-contrast imaging**

- Please specify why you're not comfortable with only performing 3D FSPGR/MPRAGE imaging post-contrast:

**Diffusion weighted imaging (DWI)**

- Which best describes your current practice in assessing glioma diffusion?

**No DWI**

- Why are you not using DWI?

**Advanced analysis**

- Please specify what analysis you use for DWI:

**DWI: b-values**

- Which b-value(s) do you use for glioma imaging?

**Perfusion MRI**

- Do you use perfusion MRI?

**No perfusion MRI**

- Why are you not using perfusion MRI?

**Routine perfusion MRI**

- Why are you using perfusion MRI routinely (rather than only upon indication)?

**Indications for perfusion MRI**

- Why do you use perfusion MRI?
- What clinical diagnoses do you use perfusion MRI for?
- How does perfusion MRI impact patient management in your practice?

**Perfusion MRI sequence**

- How long does your perfusion MRI sequence(s) last?
- Which perfusion MRI sequence(s) do you typically perform for glioma diagnosis and/or follow-up?
- Do you use a preload bolus?

**Preload bolus**

- What is your preload bolus dose?

**Total contrast dose**

- What is the total amount of contrast you use?

**Perfusion MRI assessment**

- Which perfusion analysis software do you typically use?
- How do you typically assess primary brain tumour perfusion?
- Who usually performs the perfusion post-processing?
- When do you interpret perfusion MRI?

**MR spectroscopy (MRS)**

- How often do you use MR spectroscopy (MRS)?

**No MRS**

- Why are you not using MRS?

**Indications for MRS**

- Why do you use MRS?
- What clinical diagnoses do you use MRS for?
- How does MRS impact patient management in your practice?

**MRS sequence**

- Which applies typically to your current MRS practice?
- What echo time do you typically use for MRS?
- How long does your MRS protocol last?

**MRS assessment**

- Who usually takes part in post-processing the MRS data?
- When do you interpret perfusion MRS?

**Functional MRI (fMRI)**

- Do you perform fMRI in your current practice?

**No fMRI**

- Why are you not using fMRI?

**fMRI indications and protocol**

- Why do you use fMRI?
- How does fMRI impact patient management in your practice?
- Which function(s) do you assess with fMRI (depending on lesion localisation)?
- How long does your fMRI protocol last?

**fMRI assessment**

- Who usually performs the fMRI post-processing?
- When do you interpret fMRI?
- Are the fMRI results imported in the neuronavigation system?

**Diffusion tensor imaging (DTI)**

- Do you perform DTI in your current practice?

**No DTI**

- Why are you not using DTI?

**DTI indications**

- Why do you use DTI?
- How does DTI impact patient management in your practice?
- Which do you find relevant in your current practice (depending on lesion location)?

**DTI protocol**

- How long does your DTI sequence last?
- How many directions do you typically acquire?

**DTI assessment**

- Who usually performs the DTI post-processing?
- When do you interpret DTI?
- Are the DTI results imported in the neuronavigation system?

**Treatment effects**

- Which do you typically use to distinguish tumour progression from radiation necrosis?

**Reporting**

- Do you use structured reporting/a reporting template?
- Which best describes your glioma follow-up reporting practice?

**Post-surgical imaging**

- Do you perform early (<72h) postsurgical imaging to assess extent of glioma resection?

**Post-surgical assessment**

- Do you provide a percent measure to describe completeness of resection?

**Percent resection**

- Please specify how you determine the percent completeness of resection:

**Other specialties**

- At your institution, are clinicians involved in MRI protocol development?

**Clinician involvement**

- Please specify how clinicians are involved in MRI protocol development:

**Avoid duplicate information**

All information will be kept anonymous. However, we ask you to provide the name of your institution to avoid duplicate information from the same institution. This will be kept entirely confidential and will not be included in the survey results or reporting*.

* Alternative option included ‘I confirm that I am the only person answering from my institution’.

**Institution name**

- Please provide the name of your institution:

**Final comments**

- Thank you very much for your participation. Please provide any additional comments below:
