# Supplementary material for: Glioma imaging in Europe: A survey of 220 centres and recommendations for best clinical practice
Source: Eur Radiol. 2018 Mar 13;28(8):3306–17. doi: 10.1007/s00330-018-5314-5 (PMC6028837; doi:10.1007/s00330-018-5314-5)
Supplement: Supplementary file 2 — (PDF 149 kb) [file 330_2018_5314_MOESM2_ESM.pdf]

Supplement 2 – Tables 1 to 10

General information

Institution			Position held			
Academic Hospital	General hospital	Oncology Centre	Neuro-radiologist	General radiologist	Trainee radiologist	Other profession
58.7 *(129)*	37.9 *(83)*	14.6 *(32)*	80 *(176)*	9.5 *(21)*	5.9 *(13)*	4.5 *(10)*

Hospital services			Physicist support		
Neurosurgery	Radiotherapy	Neuro-oncology	None	General physicist	Neurophysicist
84.5 *(186)*	81.4 *(179)*	76.4 *(168)*	39.5 *(87)*	35.5 *(78)*	23.6 *(52)*

Table 1 Demographic data and hospital services at participating institutions. Results are shown as % of answers *(absolute number of answers)*.

Structural MRI

Glioma primary diagnosis (MRI per week)			Glioma follow up (MRI per week)		
< 1 study	1-5 studies	> 5 studies	< 5 studies	5-10 studies	> 10 studies
16.4 *(36)*	54.1 *(119)*	28.6 *(63)*	26.4 *(58)*	34.5 *(76)*	37.3 *(82)*

Protocol duration (in minutes)				Routine structural sequences					
< 20	20-30	31-60	> 60	T2w	FLAIR	T1w	T1w+C	DWI	T2*/SWI
5 *(11)*	45.9 *(101)*	46.4 *(102)*	2.7 *(6)*	95.5 *(210)*	98.6 *(217)*	99.1 *(218)*	99.1 *(218)*	99.1 *(218)*	65 *(143)*

Table 2 Structural imaging practices in Europe. Results are shown as % of answers *(absolute number of answers)*.

Volumetric Imaging

Which 3D sequences do you use?					2D only users wishing 3D acquisition				
T2w	FLAIR	T1w	T1w+C	T2*/SWI	T2w	FLAIR	T1w	T2*/SWI	Other
3.9 *(7)*	31.1 *(56)*	9.4 *(71)*	98.3 *(177)*	15.6 *(28)*	30.8 *(8)*	65.4 *(17)*	69.2 *(18)*	15.4 *(4)*	19.2 *(5)*

T1w sequence(s) used to depict enhancement				Comfortable with FSPGR/MPRAGE only?		
2D-SE	FSPGR/ MPRAGE	3D-SE	Other	Yes	No	Don't know
52 *(111)*	72.2 *(159)*	12.8 *(28)*	8.8 *(20)*	40.5 *(89)*	44.5 *(98)*	15 *(30)*

Table 3 Volumetric imaging. Results are shown as % of answers *(absolute number of answers)*.

Diffusion-weighted MRI

Assessment of ADC map		
Visual only	ROI comparison	Advanced analysis
78.2 *(172)*	17.7 *(39)*	3.2 *(7)*

Table 4 Respondents' practices for ADC assessment. Results are shown as % of answers *(absolute number of answers)*.

Perfusion MRI (pMRI)

When do you acquire pMRI?			
Always in glioma	Primary diagnosis only	Follow up only	Upon indication only
49.1 *(108)*	10.9 *(24)*	3.6 *(8)*	21.4 *(47)*

Reasons for using pMRI				pMRI protocol duration		
For clinical diagnosis	Biopsy guidance	To guide therapy	Mainly research	≤5 mins	≤2 mins	Don't know
79.7 *(149)*	46 *(86)*	61.5 *(115)*	13.4 *(25)*	83.5 *(71)*	45 *(38)*	5 *(4)*

Reasons for always acquiring pMRI	
I want it to be available when I need it	43.6 *(61)*
I (almost) always find it useful	55.7 *(78)*
It impacts patient care and management	56.4 *(79)*
Clinicians always want it	7.9 *(11)*
I acquire it for logistical reasons (e.g. standardised protocols)	30 *(42)*
For research purposes	24.3 *(34)*
To maintain radiographers' level of experience	15.7 *(22)*

pMRI method				Preload		
DSC	DCE	ASL	≥ 2 methods	Yes	No	Don't know
81.8 *(153)*	29.4 *(55)*	12.3 *(23)*	21.4 *(40)*	46.5 *(87)*	46 *(86)*	7.5 *(14)*

Preload bolus size (of typical contrast dose)					Total contrast given (of typical contrast dose)				
1/3	½	Full	Don't know	Other	Single	1+1/3	1+1/2	Double	Don't know
42.5 *(37)*	16.1 *(14)*	10.3 *(9)*	11.5 *(10)*	19.5 *(17)*	53.8 *(99)*	13 *(24)*	8.2 *(15)*	14.7 *(27)*	7.6 *(14)*

pMRI analysis				How do you assess glioma perfusion?		
Scanner software	NordicICE	Olea	Other	Qualitatively only	ROI comparison NAWM	Other technique
78.5 *(146)*	4.8 *(9)*	4.8 *(9)*	7 *(13)*	43.5 *(81)*	51.1 *(95)*	5.4 *(10)*

Table 5 Perfusion MRI (pMRI). Results are shown as % of answers *(absolute number of answers)*.

**MR spectroscopy (MRS)**			
**When do you acquire MRS?**			
Always in glioma	Primary diagnosis only	Follow up only	Upon indication only
21 *(46)*	22.4 *(49)*	1.8 *(4)*	35.2 *(77)*

**Reasons for using MRS**				**MRS protocol duration**		
For clinical diagnosis	Biopsy guidance	To guide management	Mainly research	≤ 15 mins	≤10 mins	Don't know
87.5 *(154)*	26.7 *(47)*	46.6 *(82)*	10.8 *(19)*	75.9% *(60)*	59.5 *(47)*	10.1 *(8)*

**MRS method**		**TE**		
SVS	MVS/CSI	Short (30 ms)	Intermediate (144 ms)	Long (270 ms)
75 (132)	60.8 *(107)*	68.8 *(119)*	67.1 *(117)*	13.3 *(23)*

Table 6 MRS in glioma imaging. Results are shown as % of answers *(absolute number of answers)*. Comment: In free text answers, approximately one third of users expressed doubts about the clinical value and impact of MRS, whereas others reported a moderate to high impact. MRS reporting was most commonly undertaken together with structural MRI reporting.

**Functional MRI (fMRI)**					
**Do you use fMRI?**			**Why do you use fMRI?**		
Yes	No	Import into navigation	Pre-surgical	Guide management	Research
49.8 *(109)*	50.2 *(110)*	62.4 *(68)*	95.4 *(104)*	16.5 *(18)*	15.6 *(17)*

**fMRI protocol duration**				**Which function(s) do you assess with fMRI (depending on lesion site?)**			
< 15 mins	15-30 mins	>30 mins	Don't know	Language lateralisation	Language localisation	Motor cortex localisation	Visual cortex localisation
45.1 *(23)*	21.6 *(11)*	23.5 (12)	9.8 *(5)*	90.8 *(99)*	79.8 *(87)*	97.2 *(106)*	42.4 *(46)*

Table 7 fMRI. Results are shown as % of answers *(absolute number of answers)*

**Diffusion tensor imaging (DTI)**					
**Do you use DTI?**			**Why do you use DTI?**		
Yes	No	Import into navigation	Pre-surgical	Guide management	Research
63.9 *(140)*	36.1 *(79)*	59.3 *(83)*	87.9 *(123)*	22.1 *(31)*	20.0 *(28)*

**DTI protocol duration**		**Which function(s) do you assess with DTI (depending on lesion site?)**						
≤10 mins	Don't know	Cortico-spinal tract	Arcuate Fasciculus	Optic radiation	IFOF	Uncinate fasciculus	FA maps	Other
92.5 (50)	11.1 *(6)*	94.2 (131)	61.2 *(85)*	54.0 *(75)*	25.9 *(36)*	28.8 *(40)*	48.9 *(68)*	3.6 *(5)*

Table 8 DTI. Results are shown as % of answers *(absolute number of answers).*

Specific clinical situations								
**Used for tumour progression vs. radiation necrosis**					**Glioma follow up assessment**			
pMRI	Structural	MRS	DWI	Other	Qualitative	RANO	Volumetric	Segmentation
55.7 *(122)*	20.1 *(44)*	5.9 *(13)*	4.6 *(10)*	13.7 *(30)*	60.6 *(132)*	27.1 *(59)*	7.3 *(16)*	2.8 *(6)*
**Early postoperative imaging**					**Reporting**			
< 24 hrs	< 48 hrs	< 72 hrs	CT only		Reporting a percentage of completeness of resection	Use of a reporting template	Protocol development together with clinicians	
16.1 *(35)*	33.5 *(73)*	12.4 *(72)*	7.3 *(16)*		17.2 *(28)*	23.3 *(51)*	24.2 *(53)*	

Table 9 Clinical situations. Results are shown as % of answers *(absolute number of answers)*.

**Technical**				
**MRI Post-processing**				
	pMRI	MRS	fMRI	DTI
Radiologist/radiology fellow	71.5 *(133)*	62.5 *(110)*	61.5 *(67)*	64 *(89)*
Resident in training	22.6 *(42)*	18.2 *(32)*	15.6 *(17)*	10.1 *(14)*
Technologist/radiographer	21.5 *(40)*	31.3 *(55)*	19.3 *(21)*	20.1 *(28)*
Physicist	8.1 *(15)*	20.5 *(36)*	22.9 *(25)*	22.3 *(31)*
Researcher/research fellow	5.4 *(10)*	6.3 *(11)*	15.6 *(17)*	14.4 *(20)*
Other	7 *(13)*	5.7 *(10)*	6.4 *(7)*	8.6 *(12)*
**Reasons for non-use**				
	pMRI	MRS	fMRI	DTI
I have no technical MRI facility	42 *(14)*	46.5 *(20)*	49.5 *(54)*	40.5 *(32)*
I don't know how to implement it	15.2 *(5)*	11.6 *(5)*	23.9 *(26)*	13.9 *(11)*
I have no post-processing facility	39.4 *(13)*	32.6 *(14)*	34.9 *(38)*	35.4 *(28)*
I am not trained to interpret	27.3 *(9)*	34.9 *(15)*	40.4 *(44)*	22.8 *(18)*
Clinicians do not request it	36.4 *(12)*	32.6 *(14)*	35.8 *(39)*	*32.9 (26)*
I don't find it useful	0 *(0)*	23.3 *(10)*	6.4 *(7)*	10.1 *(8)*
It is not reimbursed	15.2 *(5)*	14 *(6)*	11 *(12)*	12.7 *(10)*
There is not enough time to perform it	21.2 *(7)*	16.3 *(7)*	33.9 *(37)*	25.3 *(20)*
Other	3 *(1)*	2.3 *(1)*	11 *(12)*	7.6 *(6)*

Table 10 Advanced imaging data post-processing and reasons stated for non-use of modalities. Results are shown as % of answers *(absolute number of answers)*.

General comment: A number of questions included the option 'other'. Please note, if this was answered by few individuals (< 5%), percentages are not quoted in the results. A small numbers of free text answers could not be deciphered, so these were excluded from the analysis.
